# Mitochondrial DNA ancestry, HPV infection and the risk of cervical cancer in a multiethnic population of northeastern Argentina

**DOI:** 10.1371/journal.pone.0190966

**Published:** 2018-01-12

**Authors:** Ines Badano, Daiana J. Sanabria, Maria E. Totaro, Samara Rubinstein, Juan A. Gili, Domingo J. Liotta, Maria A. Picconi, Rodolfo H. Campos, Theodore G. Schurr

**Affiliations:** 1 Laboratorio de Biología Molecular Aplicada, Facultad de Ciencias Exactas, Químicas y Naturales, Universidad Nacional de Misiones, Misiones, Argentina; 2 Consejo Nacional de Investigaciones Científicas y Técnicas (CONICET), Buenos Aires, Argentina; 3 Laboratory of Molecular Anthropology, Department of Anthropology, University of Pennsylvania, Philadelphia, United States of America; 4 Laboratorio de Epidemiología Genética, Dirección de Investigación CEMIC-CONICET, Buenos Aires, Argentina; 5 Servicio de Virus Oncogénicos, Departamento de Virología, INEI-ANLIS “Dr. Carlos G. Malbrán”, Buenos Aires, Argentina; 6 Cátedra de Virología, Facultad de Farmacia y Bioquímica, Universidad de Buenos Aires, Buenos Aires, Argentina; Fondazione IRCCS Istituto Nazionale dei Tumori, ITALY

## Abstract

**Background:**

Misiones Province in northeastern Argentina is considered to be a region with a high prevalence of HPV infection and a high mortality rate due to cervical cancer. The reasons for this epidemiological trend are not completely understood. To gain insight into this problem, we explored the relationship between mitochondrial DNA (mtDNA) ancestry, HPV infection, and development of cervical lesions/cancer in women from the city of Posadas in Misiones Province.

**Methods:**

Two hundred and sixty-one women, including 92 cases of patients diagnosed with cervical lesions and 169 controls, were analyzed. mtDNA ancestry was assessed through HVS1 sequencing, while the detection and typing of HPV infection was conducted through nested multiplex PCR analysis. Multivariate logistic regression was conducted with the resulting data to estimate the odds ratios (ORs) adjusted by socio-demographic variables.

**Results:**

The study participants showed 68.6% Amerindian, 26.1% European and 5.3% African mtDNA ancestry, respectively. Multiple regression analysis showed that women with African mtDNAs were three times more likely to develop a cervical lesion than those with Native American or European mtDNAs [OR of 3.8 (1.2–11.5) for ancestry and OR of 3.5 (1.0–12.0) for L haplogroups], although the associated p values were not significant when tested under more complex multivariate models. HPV infection and the development of cervical lesions/cancer were significant for all tested models, with the highest OR values for HPV16 [OR of 24.2 (9.3–62.7)] and HPV-58 [OR of 19.0 (2.4–147.7)].

**Conclusion:**

HPV infection remains a central risk factor for cervical cancer in the Posadas population. The potential role of African mtDNA ancestry opens a new avenue for future medical association studies in multiethnic populations, and will require further confirmation in large-scale studies.

## Introduction

The human papillomavirus (HPV) is a small, nonenveloped virus with a circular double-stranded DNA genome (of approximately 8 kb) that belongs to the family *Papillomaviridae* [1]. It infects stratified squamous epithelia (mucosal and cutaneous), where it can cause neoplasias or persist asymptomatically. In particular, HPV genital infections by certain types (identified as high-risk) can lead to the development of cervical lesions and cervical cancer [2,3]. Currently, more than sixteen HPV strains are identified as high-risk types (HPV-HR) for the development of cervical cancer, and are classified as Genus *Alpha-Papillomavirus*, species *A6*, *A7* and *A9* [1–3].

Cervical cancer is the second most common female cancer in women aged 15 to 44 from across the world. Incidence rates vary within different geographic regions, being higher in Africa than in Europe (27.6 cases per 100,000 vs. 11.4/100,000), and intermediate in the Americas and Asia (14.9/100,000 and 12.7/100,000 respectively) [4]. These differences have been attributed to the socio-cultural characteristics of the populations in these regions and the lack of effective screening programs [4]. However, the genetic background of the host population may further influence the rate of cervical cancer [5–9].

Since the early 1990s, epidemiological studies addressing the role of “ethnicity” (i.e., genetic ancestry) in the development of cervical cancer have indicated that African-Americans and Amerindians (“Latinos or Hispanics”) from the United States are at a higher risk of developing cervical cancer than “whites” [10, 11]. However, these studies classified populations based on self-reported origin, surname or skin color [10], characteristics that have been shown to be poor markers of genetic ancestry in diverse populations of Latin America such as those from Brazil and Argentina [12, 13]. Moreover, after adjustment by socio-economic status, such associations tend to disappear, indicating that social factors (rather than ethnicity or ancestry) have a more important influence on cervical cancer incidence [10, 14]. For these reasons, the potential influence of genetic ancestry on the prevalence of cervical cancer within different populations has not yet been comprehensively addressed.

The analysis of mitochondrial DNA (mtDNA) sequence variation can be used to assess the maternal genetic ancestry of an individual [15]. The mtDNA is a 16,569-base-pair circular double-stranded molecule containing 37 genes that play an important role in cellular energy production and apoptosis [16]. An individual’s mtDNA can be placed in a haplogroup (maternal lineage) based on the set of polymorphisms or single nucleotide polymorphisms (SNPs) present in its sequence. Based on numerous studies, it is now clear that many haplogroups originated in specific areas of the world and, thus, predominate in local populations. For example, four lineages (L0, L1, L2, and L3) are common in African and African-derived populations [17], nine (H, I, J, K, T, U, V, W and X) in European populations [18], and eight (A, B, C, D, E, F, G and M) in Asian populations [19, 20], with four Asian-derived haplogroups (A2, B2, D1 and C1) being specific to Native Americans [21, 22].

In addition to their phylogeographic histories, there is growing interest in the relationship between mtDNA haplogroups and the development of various types of female cancers. The presence of the Amerindian haplogroup D5 has been associated with breast cancer [23], the European haplogroup UK with vulvar cancer [24], and the Amerindian haplogroup B2 with cervical cancer [25]. These findings suggest that maternal genetic ancestry could play a role in the incidence of these diseases.

Similarly, an understanding of matrilineal ancestry is important for assessing disease prevalence and susceptibility in Argentina. In Argentina, a large proportion of the population has Native American ancestry (45% and 70% for the central and northern areas, respectively) followed by European (50 to 29%) and African (<3%) ancestry [26, 27]. Nevertheless, the effect of mitochondrial genetic ancestry on cervical cancer in this country is not currently known.

This is also the case for Misiones Province, which is located in the northeastern tip of Argentina and shares international borders with Paraguay and Brazil. From an epidemiological point of view, this province is considered to be a region with a high prevalence of HPV infection and mortality rate of cervical carcinoma (33–43% and 12/100,000 individuals, respectively) compared to other areas of the country, such as Buenos Aires (30% and 3/100,000) [28, 29]. Its current population is also the product of generations of intermixing between various groups, including Native Americans, who originally inhabited this part of South America, Spanish conquerors, and a large European immigrant population that arrived in the 1930s [30]. Further admixture has occurred through recent immigration from bordering South American countries such as Brazil and Paraguay [30].

Despite this complex history, the genetic background of this population has been poorly studied [31–33]. Moreover, the genetic influences on HPV infection and cervical carcinoma incidence in the region have not been examined. Therefore, the objective of this study was to explore the relationship between mtDNA ancestry, HPV infection and the development of cervical lesions and cancer in women from this multiethnic region of northeastern Argentina.

## Materials and methods

### Ethic statement

This study was conducted with the approval of The Ethics Committee of the Dr. Ramón Madariaga Hospital, Posadas, Argentina (Departamento de Docencia e Investigación, Comité de Bioética, Hospital Dr. Ramón Madariaga, Posadas, Misiones). All experiments were performed in compliance with institutional guidelines and in accordance with ethical standards of the Declaration of Helsinski.

### Study design

We conducted a retrospective study of genetic risk factors involved in the development of cervical lesions and cancer in women from Posadas in Misiones Province. The study samples were obtained from women attending to different health centers and clinics in the city between 2005–14. The study samples were archived at the Laboratorio de Biología Molecular Aplicada (LaBiMAp) of the Facultad de Ciencias Exactas, Químicas y Naturales of the University of Misiones. For each sample, a database containing information about participant age, Pap cytology, date and the location of the sampling center, healthcare system, nationality and place of residence was recorded. Information about the HPV status of the participants was retrieved from the database (n = 128) or assessed in this study (n = 136) (see the “HPV detection and typing” section below).

All samples from the LaBiMAp were obtained with informed consent in the context of previous epidemiological studies, with none of them addressing mtDNA characterization [8,29]. The Ethics Committee of the Dr. Ramón Madariaga Hospital, Posadas, Argentina, approved the use of these biological samples for this study.

### Population demographics

Posadas is the capital of Misiones Province and currently has a population of 324,758 people [34]. Of these, 106,141 are women of reproductive age (more than 19 years old) [34], with the mortality rate for cervical cancer being 12/100,000 [28]. To date, no systematic survey of genetic diversity in the inhabitants of this city has been undertaken, and the possible existence of association between socio-demographic variables and mtDNA ancestry is unknown. Such associations have been reported for other Latin-American populations, such as that of Uruguay [13, 35], and their existence should be addressed, since they could affect the outcome of genetic association studies [35].

In this study, samples were recruited from health centers located at three locations within the city, including the Downtown area and the 1^st^ and 2^nd^ Urban Belts. Socio-demographic variables from each location were not recorded although, based on our own records, we can briefly describe them as follows. The Downtown area is characterized by private practices, with the resident population being of upper-middle income status and having health insurance. By contrast, the 1^st^ and 2^nd^ Urban Belts contain the main city hospital and a community health center (respectively). Both are public health centers with an attending population of middle-low income status whose members largely lack health insurance. To account for the potential effect of sample center location, those sociodemographic variables showing associations with mtDNA ancestry were included in the multivariate analysis of this study (see the “Association Analysis” section).

### Nomenclature

The cytological classifications used in this study were as follows: NILM: negative for intraepithelial lesion and malignancy; L-SIL: low-grade squamous intraepithelial lesion; H-SIL: high-grade squamous intraepithelial lesion; CIS: carcinoma *in situ*; and ISCC: invasive squamous cell carcinoma [36]. The notation H-SIL+ was used to group H-SIL + CIS + ISCC patients.

### Study groups

In this study, 134 patients diagnosed with cervical lesions (60 L-SIL, 59 H-SIL and 15 with cervical cancer *in situ/invasive)* were selected without personal identifiers from the laboratory registry. They represented about 80% of the patients recorded as HSIL+ for the period between 2005–14. A total of 186 control subjects were selected from the same database, with all of their samples being acquired during the same period as those of the case patients under conditions of anonymity. DNAs for the Controls and L-SIL samples were obtained from cervical scrapes, whereas those for the H-SIL+ group were extracted from biopsies of formalin-fixed, paraffin-embedded tissues (fixed biopsies). Although the initial sample size was 320 individuals, complete genotyping to estimate mtDNA ancestry was successful in 261 of them (81.6%), which comprises the final number analyzed in this study (see the “Mitochondrial DNA Analysis” section).

### Mitochondrial DNA analysis

mtDNA haplotypes were defined through direct sequencing of the HVS1 region of the control region (np 16024–16383). Briefly, the HVS1 was PCR amplified using primers 15977-F (5´-CCA CCA TTA GCA CCC AAA GC-3´) and 16552-R (5´-TAA GGG GAA CGT GTG GGC-3´). Positive amplicons were purified using commercial kits (ADN PuriPrep-GP kit, Inbio Highway) and directly sequenced using the original primers through sequencing services using a Beckman Coulter CEQ 2000XL DNA Analysis System (Cromatida, Argentina). A total of 320 samples were processed and 280 were positive for mtDNA (87.5%). Of these, quality sequences were obtained from 261 samples (93.2%). The mtDNA sequences were edited and aligned using Codon Code aligner software v 3.0.1 (CodonCode Corporation). HVS1 haplotypes were classified into haplogroups by using HaploGrep and Phylotree build 17.0 [37, 38]. mtDNA haplotypes and their haplogroup status are shown in S1 Table. HVS-1 sequences were deposited in GenBank under accession numbers: KY344532—KY344740.

### Human papillomavirus detection and typing

HPV detection was performed using PCR amplification with L1 consensus primers MY09–MY11 [39]. HPV DNA-positive samples were typed by E6-Nested Multiplex PCR (E6-NMPX) with cocktails of primers C-1 (high-risk HPVs 16, 18, 31, 45, and 59) and C-2 (high-risk HPVs 33, 56, 52, 58 and low-risk HPVs 6 and 11) [40] to determine the type of strain(s) present in the samples. Positive samples that were not revealed as positive by E6-NMPX amplification were left as “HPV undetermined”.

### Association analysis

The distribution of mtDNA haplogroups or HPV types between study groups was compared in contingency tables by using χ^2^ or two-tailed Fisher exact test for cell counts below five. Similarly, the distribution of mtDNA haplogroups according to socio-demographic variables (age, sample center location, healthcare system, nationality and place of residence) was tested in contingency tables (χ^2^ or two-tailed Fisher exact test), with the exception of age, which was tested using the Median test. Those variables found to be significant were included in the multivariate analysis.

Multivariate ordinal logistic regression was used to estimate the OR and 95% confidence intervals (CIs). For these tests, each variable was classified as follows: (1) *Cytological diagnosis*, including three categories of progressive nature [NLIM, LSIL and HSIL+]; (2) *mtDNA ancestry*, including three categories [Amerindian, European and African]; (3) *mtDNA haplogroups*, which included eight categories (A, B, C, D, HV, JT, UK, L), with haplogroups occurring at a frequency less than 5% being “clustered” into larger monophyletic clades such as UK, JT, HV and L (L0, L1, L2 and L3) called “phylogroups”; and (4) *HPV types*, including 10 categories [HPV negative, HPV undetermined, HPV6/11, HPV56, HPV58, Multiple Infections, HPV52, HPV33, other HPV-HR and HPV16], in which those HPVs occurring at frequencies less than 5% were clustered into larger groups (other HPV-HR, including species *A7*: 18, 45 and 59). For the analysis, HPV types were tested by ordering them according to their increase in the OR according to Muñoz et al., 2003 [3]. The final models of analysis included (I) Pap cytology and HPV infection, (II) Pap cytology and mtDNA ancestry, adjusted by socio-demographic variables, (III) Pap cytology and mtDNA haplogroups, adjusted by socio-demographic variables, (IV) Pap cytology, mtDNA ancestry and HPV infection, adjusted by socio-demographic variables, and (V) Pap cytology, mtDNA haplogroups and HPV infection, adjusted by socio-demographic variables. Ordered logistic regression produced a unified OR value, taking under consideration the three progressive stages of Pap cytology. All calculations were made using Stata 14.0 (StataCorp LLC, Texas, USA, 2015). Forest plots were made using GraphPad Prism version 7.0d for Mac OS X (GraphPad Software Inc, La Jolla California USA, www.graphpad.com).

## Results

### Study groups

Two hundred and sixty one samples, including 92 cases of patients diagnosed with cervical lesions (50 L-SIL, 35 H-SIL and 7 cervical cancer in situ/invasive) and 169 control subjects were analyzed. The average age of the H-SIL+ cases was 32.1 years (age range 20–54 years), that of the L-SIL cases was 31.5 years (20–50 years), and that of controls was 36.0 years (10–83 years).

### Human papillomavirus typing distribution and cervical lesions

A total of 130 samples were identified as positive for HPV (49.8%) and 11 different viral types (16, 18, 31, 33, 45, 52, 56, 58, 59, 6, 11) were identified in them. HPV infection was higher in women with cervical lesions (70%) compared to those without lesions (37.9%), a pattern consistent with its role in cervical cancer development. Briefly, HPV16 infection had the highest frequency among study groups (9.5% of NILM; 22% of L-SILs and 57.1% of H-SIL+), followed by HPV6/11 (3.0% of NILM; 20% of L-SILs and 16.7% of H-SIL+). Multiple infections (double and triple) were present in 11.1% of women while 8% of the viral infections remained untyped. Details about HPV types and their frequencies are shown in Table 1.

**Table 1:** Frequency of HPV types in the study groups.

	NLIM (169)	LSIL (50)	HSIL+ (42)	Total (261)	*p* value^a^
**HPV positive**	**64 (37.9)**	**35 (70.0)**	**31 (73.8)**	**130 (49.8)**	**< 0.001**
HPV Negative	105 (62.1)	15 (30.0)	11 (26.2)	131 (50.2)	
**HPV types**^**b**^					
**16**	**16 (9.5)**	**11 (22.0)**	**24 (57.1)**	**51 (19.5)**	**< 0.001**
**18**	**1 (0.6)**	**5 (10.0)**	**0 (0.0)**	**6 (2.3)**	**< 0.001**
31	4 (2.4)	3 (6.0)	2 (4.8)	9 (3.4)	0.409
33	10 (5.9)	3 (6.0)	1 (2.4)	14 (5.4)	0.645
45	3 (1.8)	0 (0.0)	0 (0.0)	3 (1.1)	0.438
52	8 (4.7)	2 (4.0)	1 (2.4)	11 (4.2)	0.791
56	13 (7.7)	4 (8.0)	1 (2.4)	18 (6.9)	0.450
58	2 (1.2)	2 (4.0)	2 (4.8)	6 (2.3)	0.257
**59**	**1 (0.6)**	**3 (6.0)**	**0 (0.0)**	**4 (1.5)**	**0.016**
**6/11**	**5 (3.0)**	**10 (20.0)**	**11 (16.7)**	**26 (8.4)**	**< 0.001**
**Multiple-Infections**	**13 (7.7)**	**10 (20.0)**	**6 (14.3)**	**29 (11.1)**	**> 0.040**
HPV-Undetermined	16 (9.5)	5 (10.0)	0 (0.0)	21 (8.0)	> 0.111

^a^*p* values for the distribution in a contingency tables (χ^2^ or fisher exact test). Significant *p* values are shown in **boldface.**

^b^HPV infection counts and frequencies include types in single and multiple infections.

The results of the association analysis are shown in Table 2. The highest OR values were found for HPV16 [OR = 18.3 (7.6–44.3)], HPV58 [OR = 13.8 (2.0–95.4)] and HPV6/11 [OR = 9.3 (2.9–30.0)]. Other significant associations included HPV6/11 [OR = 9.3 (2.9–30.0)] and other HPV-HR [OR = 5.9 (1.5–22.1)].

**Table 2:** Association analysis between HPV infection and development of cervical lesions.

HPV	O.R.	CI 95%	*p* value
HPV negative	1	Ref	
**HPV positive**	**4.0**	**2.3–6.9**	**<0.001**
HPV types^a^			
**16**	**18.3**	**7.6–44.3**	**<0.001**
33	1.3	0.3–5.1	0.696
52	0.6	0.1–5.3	0.667
56	1.5	0.4–5.9	0.582
**58**	**13.8**	**2.0–95.4**	**0.008**
**6/11**	**9.3**	**2.9–30.0**	**<0.001**
**Others HPV-HR**^**b**^	**5.9**	**1.5–22.1**	**0.009**
Multiple-Infections	**4.5**	**2.0–10.0**	**<0.001**
HPV-Undetermined	1.1	0.4–3.3	0.840

^a^Ordered logistic regression for Pap cytology and HPV infection (Model I). Significant associations are shown in **boldface.**

^b^Including: HPV18, HPV59, HPV45 (specie *A7*).

### Mitochondrial DNA ancestry and socio-demographic variables

The mtDNA ancestry of the study population was 68.6% Amerindian, 26.1% European and 5.3% African in origin, respectively. There were statistically significant differences in mtDNA ancestry among the study groups, with women having European mtDNAs being more frequently found in the cervical lesion groups (L-SIL and H-SIL+) than those women with Amerindian mtDNAs (p<0.05). In addition, we explored the effect of socio-demographic variables on the mtDNA distribution within this population (Table 3). Notably, mtDNA ancestry was associated with sample center location, healthcare system and nationality, but not with place of residence or age. Based on these results, these significant variables were included in a multivariate analysis.

**Table 3:** mtDNA ancestry, cervical lesions and socio-demographic variables for the Posadas population.

		Amerindian (n = 180)	European (n = 67)	African (n = 14)	*p* value^a^
Pap cytology	NILM	130	32	7	
	LSIL	30	18	2	**0.002**
	HSIL+	20	17	5	
Age	Mean	35.1	34.3	32.1	
	Median	32.5	33.0	32.0	0.619^b^
	SD	11.3	9.3	5.6	
	Range	18–83	20–66	22–38	
Sample Location	Downtown	54	51	4	
	1^st^ Urban Belt	32	8	3	**<0.001**
	2^nd^ Urban Belt	94	8	7	
Nationality	Argentinian	169	66	11	
	Others	11	1	3	**0.013**
Place of Living	Posadas	166	62	11	
	Countryside	3	4	0	0.175
HealthCare System	Public	126	16	10	
	Private	54	51	4	**<0.001**

Note: Some columns do not add up to the total because of missing data.

^a^*p* values for the distribution in a contingency tables (χ^2^ or fisher exact test). Significant *p* values are shown in **boldface**.

^b^*p* value for the Median Test.

### Mitochondrial DNA haplogroups and cervical lesions

We identified 17 different mtDNA haplogroups in the study population, all of which corresponded to the major maternal lineages that have contributed to Argentinean history. These included A2, B2, C1, D1 (Amerindian), H, HV, I, J, K, T, U, V, X2 (European) and L0, L1, L2, L3 (African) (S1 Table). mtDNA haplogroups (or phylogroups) with a frequency >5% are shown in Table 4. There were statistically significant differences in the distribution of European phylogroup JT, which occurred more frequently in the cervical lesions groups (p<0.05). However, this phylogroup was not found to be associated with the development of cervical lesions in the adjusted multivariate analysis.

**Table 4:** Frequency of mtDNA haplogroups and phylogroups in the study groups.

	NLIM (169)	LSIL (50)	HSIL+ (42)	TOTAL (261)	*p* value^a^
A	37 (21.9)	9 (18.0)	7 (16.7)	53 (20.3)	0.680
B	26 (15.4)	5 (10.0)	6 (14.3)	37 (14.2)	0.631
C	45 (26.6)	11 (22.0)	4 (9.5)	60 (23.0)	0.061
D	21 (12.4)	4 (8.0)	3 (7.1)	28 (10.7)	0.482
Subtotal Amerindian	**130 (76.9)**	**30 (60.0)**	**20 (47.6)**	**180 (69.0)**	**<0.001**
HV	20 (11.8)	10 (20.0)	7 (16.7)	37 (14.2)	0.306
JT	**3 (1.8)**	**4 (8.0)**	**5 (11.9)**	**12 (4.6)**	**0.009**
UK	8 (4.7)	3 (6.0)	4 (9.5)	15 (5.7)	0.489
**Subtotal European**	**32 (18.9)**	**18 (36.0)**	**17 (40.5)**	**67 (25.7)**	**0.003**
Subtotal African^b^	7 (4.1)	2 (4.0)	5 (11.9)	14 (5.4)	0.121
Others^c^	2 (1.2)	2 (4.0)	1 (2.4)	5 (1.9)	0.307
Total	169 (100.0)	50 (100.0)	42 (100.0)	261 (100.0)	

^a^ *p* values for the distribution in a contingency tables (χ^2^ or fisher exact test). Significant *p* values are shown in **boldface**.

^b^Haplogroups L0, L1, L2 and L3.

^c^ Haplogroups I, W and X.

### Multivariate logistic regression models

**Fig 1:**
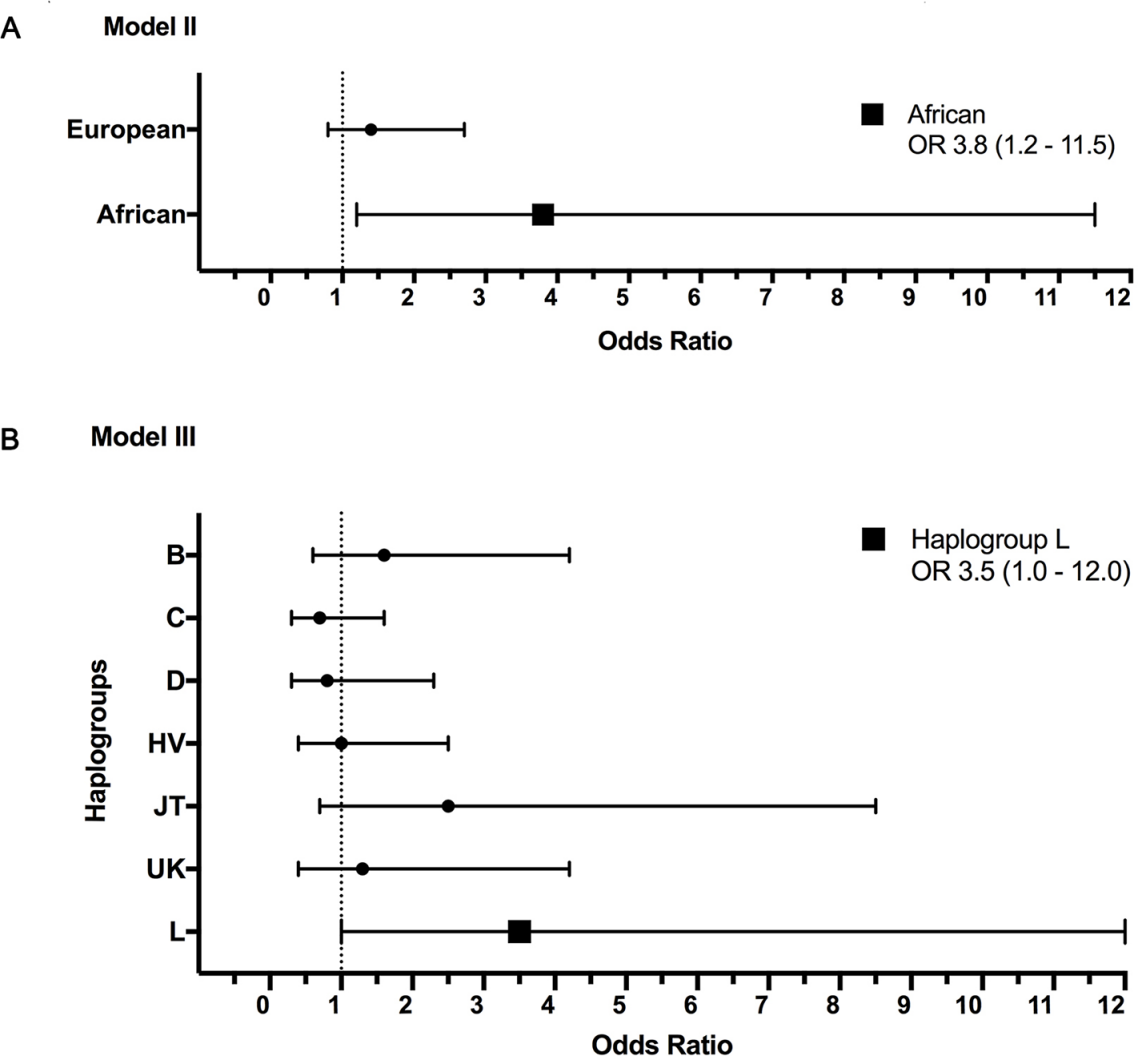
Multivariate regression analysis for mtDNA and cervical cancer. Legend: A Forest Plot showing odds ratio values and 95% confidence intervals for: (A) Model II—Pap cytology and mtDNA ancestry, adjusted by socio-demographic variables and, (B) Model III—Pap cytology and mtDNA haplogroups, adjusted by socio-demographic variables. The x-axis represents the odds ratio (circles and square) and 95% confidence intervals (whiskers). The dashed vertical line indicates an OR value of 1. Non-significant values are shown as a small circle. Significant values are shown as square, with the OR (CI95%) as a legend into the figure. Details on other OR (CI = 95%) and p values are provided in the Supplementary Information (S2 and S3 Tables). Amerindian ancestry and haplogroup A were not plotted since they were used as reference values (OR = 1).

The association between mtDNA ancestry and cervical lesion development was estimated using logistic regression models adjusted by significant socio-demographic variables (sample center location, healthcare system and nationality). The results are shown in Fig 1. In this population, women with African maternal ancestry were more likely to develop a cervical lesion than those having Amerindian or European maternal ancestry, with an OR of 3.8 (1.2–11.5) and a p value of 0.018. This association remained positive at the level of phylogroup with an OR of 3.5 (1.0–12.0) for haplogroup L, although the significance of this finding was weak, with a p value of 0.043. Details about the OR estimates are provided in S2 and S3 Tables.

**Fig 2:**
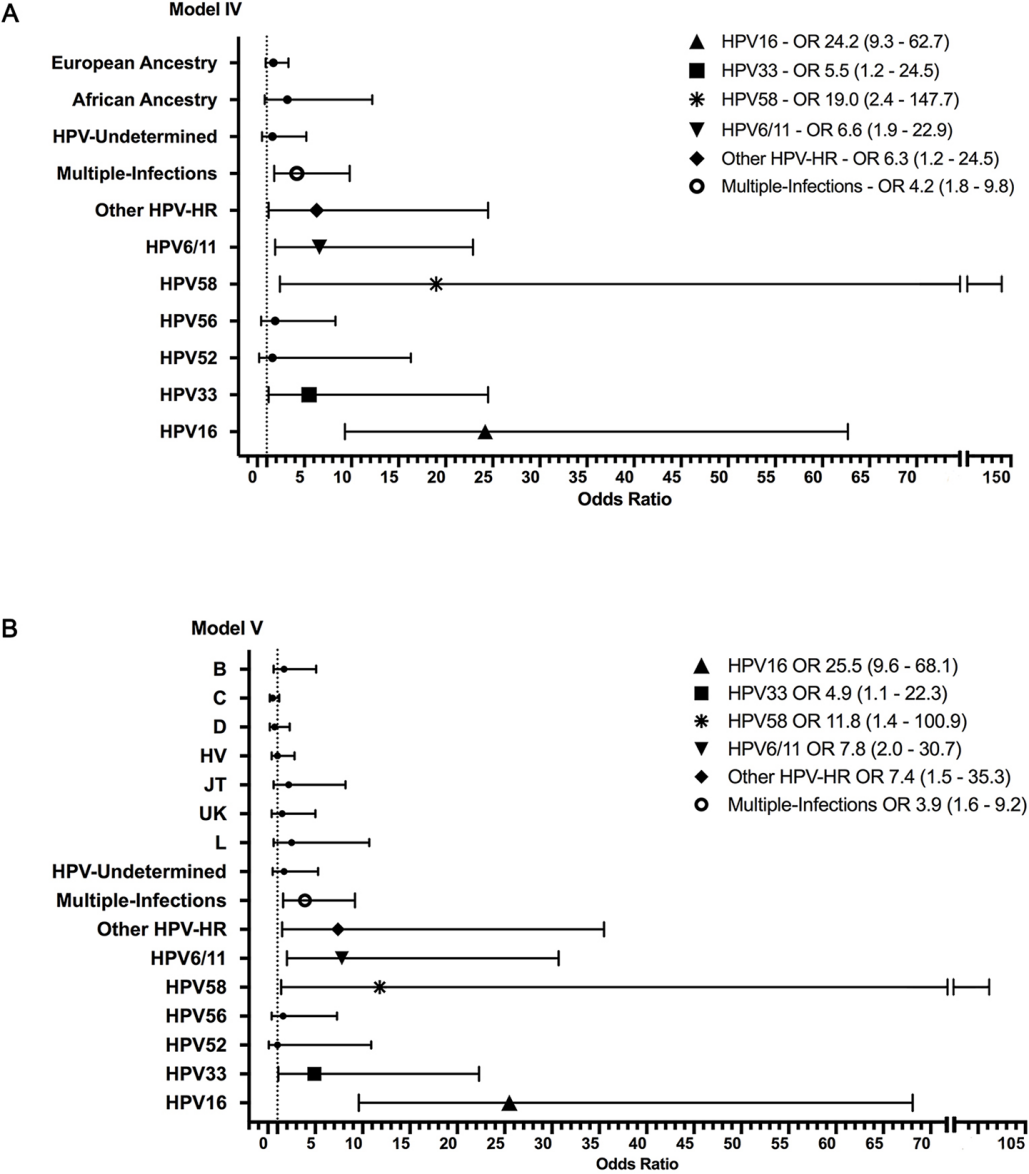
Multivariate regression analysis for mtDNA, HPV infection and cervical cancer. Legend: A Forest Plot showing the odds ratio values and 95% confidence intervals for: (A) Model IV—Pap cytology, mtDNA ancestry and HPV infection, adjusted by socio-demographic variables and (B) Model V—Pap cytology, mtDNA haplogroups and HPV infection, adjusted by socio-demographic variables. The x-axis represents the odds ratio (circles, square, others) and 95% confidence intervals (whiskers). The dashed vertical line indicates the OR value of 1. Non-significant values are shown as a small filled circle. Significant values are shown as squares (HPV33), diamonds (other HPV-HR), asterisks (HPV58), triangles (HPV16), inverted triangles (HPV6/11), and unfilled circles (multiple infections), with the OR (CI = 95%) as a legend into the figure. Details about other OR (CI = 95%) and p values are provided in the Supplementary Information (S4 and S5 Tables). Amerindian ancestry and haplogroup A were not plotted since they were used as a reference values.

We ran an additional test that included HPV infection in the regression models (Model IV: Pap cytology, mtDNA ethnic ancestry and HPV infection, adjusted by socio-demographic variables; and Model V: Pap cytology, mtDNA haplogroups and HPV infection, adjusted by socio-demographic variables). The results are shown in Fig 2. Both analyses showed that women carrying African mtDNAs were nearly three times more likely to develop a cervical lesion than those having Native American or European mtDNAs, with a OR of 3.2 (0.8–12.2) for Model IV and a OR of 2.5 (0.6–10.7) for Model V, although the associated p values were not significant (0.085 and 0.229 respectively). Different from mtDNA influence, the development of cervical lesion was consistently associated with HPV infection by HPV16 at an OR of 24.2 (9.3–62.7), and HPV-58 at an OR of 19.0 (2.4–147.7), among other types, across all tested models (Fig 2). Details about the OR estimates are provided in S4 and S5 Tables.

## Discussion

Multi-ethnic populations offer an opportunity to test the effects of ancestry on disease within the same population [41]. Based on historical records, the population of Posadas was a suitable candidate with which to use this approach, although its genetic composition was not well known. This study revealed considerable maternal genetic diversity in the Posadas population, with 68.6% of the participants having Amerindian, 26.1% European and 5.3% African mtDNAs, respectively. This general genetic profile is similar to that previously reported for northeastern Argentina [26, 27], but differed from the reported national average and from that of larger cities in Argentina such as La Plata and Córdoba (with an European component of nearly 50%) [42, 43]. Overall, the genetic structure within the country is an important issue for future nationwide medical studies.

Moreover, there is growing evidence that mtDNA variation is deeply structured in worldwide populations and also more susceptible to false-positive findings in association studies than autosomal SNPs [44, 45]. In this regard, we observed significant differences between the maternal lineage distribution and several socio-demographic variables (sample center location, healthcare system and nationality), a feature shared with other Latin American populations [12, 35]. Hence, controlling these variables through the use of adjusted ORs and multivariate analysis was necessary to reduce potential false positives.

In Argentina, there are no previous reports on the relationship between mtDNA ancestry and cervical cancer, and only three publications have addressed this issue in the published literature [25, 46, 47]. Among them, the risk of developing cervical cancer has been linked to Amerindian haplogroup B2 in Mexico [OR 1.6 (1.05–2.58)] [25], to Asian haplogroup M in India (OR not provided, but the marker showed a frequency of 65% in cases and 16% in controls) [47], and to Asian-American haplogroup D4 in China [OR 1.034 (1.004–1.006)] [47], but the latter value is questionable as a risk factor (usually OR >1.5). In the present study, none of those haplogroups (B2, M or D4) were identified as risk factors for the development of cervical lesions in Posadas. Instead, we observed an association with African ancestry [OR of 3.8 (1.3–11.3)] or phylogroup L [OR of 3.8 (1.3–11.3)]. However, this association did not remain significant when tested under more complex multivariate models (including HPV infection, mtDNA and cervical lesions). The latter finding adds a note of caution when interpreting African ancestry as a candidate factor for cervical cancer development. Moreover, we were actually underpowered when carrying out the test of interaction between viral and mitochondrial markers.

An additional study limitation was the unsuccessful mtDNA typing in 18.4% of the samples, a value above the usual threshold of 5–10%. The result can be attributed to the use of DNA from formalin-fixed, paraffin-embedded tissue in the group of H-SIL+ samples. This source of DNA has been reported to have a lower DNA quality/quantity compared to cervical scrapes, due to several potential problems including DNA cross-linking, DNA fragmentation and the presence of PCR inhibitors [48, 49]. Some studies have further indicated that the length of DNA fragments might be an important factor, reporting difficulties with amplicons >300 bp in size [50]. In our study, the amplicon size of the mtDNA HVS-1 was 575 bp, suggesting it may have been a limiting factor in fixed biopsies samples. Thus, future studies with improved protocols and larger sample sizes will be needed to confirm our findings.

Nevertheless, epidemiological data derived from US cancer registries indicated that African Americans, referred to as ‘black’ in them, have a higher incidence of cervical cancer and a lesser survival rate from all combined malignancies relative to individuals of the ‘white’ population [51]. These differences were historically attributed to confounding socio-economical and behavioral factors, such as diet, alcohol abuse, smoking, and access to screening and treatment. However, there is evidence that the survival disparity persists after normalization for these factors [52]. Additional epidemiological data show a better outcome in cancer patients with Hispanic versus African-American background, in spite of comparable socioeconomic status, a phenomenon known as the ‘Hispanic paradox’ [53]. Differences in lifestyles, specifically diet, have been proposed as a possible explanation for this observation [53], although genetic factors may be involved.

Our results may help to provide new working hypotheses about these risk factors. Argentina is an area of considerable interest in this respect, as blacks have historically contributed to its population but today are not phenotypically “visible” in its inhabitants [54]. As a result, it is not possible to classify an individual as having African descendant based on morphological characteristics alone [54]. This fact makes them different from African descendants from the United States, or those from other Latin American countries like Brazil or Colombia [55, 56].

As a final note, the African genetic component in this study population was higher than the nationwide average of 2% [26, 27]. This observation can be attributed to the geographic proximity of Misiones to Brazil, a country with a significant African genetic component in its population [56]. Misiones Province shares nearly 65% of its boundary with Brazil and has more than ten international crossing borders that allowed migration between both countries [57]. In addition, the origin of African descendants in Misiones can be traced back to colonial times (18^th^-19^th^ centuries), when African slaves from Brazil escaped by crossing the Uruguay River and hiding themselves in the forest of Misiones in search of freedom. In addition, the War of the Triple Alliance (1865–1870) involved soldiers of Afro-Brazilian origin, with some remaining in Posadas after the end of the war. From 1900 onward, African descendants from Brazil migrated to Misiones as a product of the dynamic socio-political and economical situation of the two bordering countries [58].

In spite of these historical records, the 2010 census indicated that only 0.3% of individuals from the Misiones population consider themselves to be African descendants [59]. However, this study has some limitations when extrapolating the patterns of genetic diversity to populations as a whole, as it only provides a view of female genetic history (as inferred from the mtDNA). In Argentina, the use of different markers (Y chromosome, AIMs, mtDNA) usually provides different proportions of geographic ancestry, while also indicating a sex-bias in the contribution coming from the different source populations [26, 43]. Nevertheless, we have shown that African genes (markers) can be traced in the Argentinean gene pool, and indicated the potential association between African ancestry and cervical cancer, emphasizing the importance of taking African genetic variation into account in future association studies.

Different from the mtDNA results, HPV infection was strongly associated with the development of cervical lesions for all the tested models, a finding that highlights the biological meaning of such an association.

## Conclusion

We have characterized the genetic ancestry of a population from Posadas in Misiones Province, northeastern Argentina, and identified a potential association between African mtDNA ancestry and cervical cancer, a result that will require future replication in an independent larger sample. HPV infection remains a central risk factor for cervical cancer in Posadas population, a finding that is of utmost importance in the vaccine era.

## Supporting information

S1 Table Mitochondrial DNA SNPs haplotypes and haplogroups. Legend: SNPs positions are denoted using the CRS sequence as reference.
(DOCX)

S2 Table Association analysis between mtDNA ancestry and cervical lesions. Legend: ^a^O.R. adjusted by sample center location and nationality (Model II). Significant associations are in **boldface.**
(DOCX)

S3 Table Association analysis between mtDNA haplogroups and cervical lesions. Legend: ^a^O.R. adjusted by sample center location and nationality (Model III). Significant associations are in **boldface.**
(DOCX)

S4 Table Association analysis between Pap cytology, mtDNA ancestry and HPV infection. Legend: ^a^O.R. adjusted by socio-demographic variables (Model IV). Significant associations are in **boldface.** ^b^Including: HPV18, HPV59, HPV45 (specie *A7*).
(DOCX)

S5 Table Association analysis between Pap cytology, mtDNA haplogroups and HPV infection. Legend: O.R. adjusted by socio-demographic variables (Model V). Significant associations are shown in **boldface.**
(DOCX)
