# Supplementary material for: Mitochondrial DNA ancestry, HPV infection and the risk of cervical cancer in a multiethnic population of northeastern Argentina
Source: PLoS One. 2018 Jan 12;13(1):e0190966. doi: 10.1371/journal.pone.0190966 (PMC5766133; doi:10.1371/journal.pone.0190966)
Supplement: S1 Table — Legend: SNPs positions are denoted using the CRS sequence as reference. (DOCX) [file pone.0190966.s001.docx]

**Supplementary Table 1: mtDNA SNPs haplotypes and haplogroups**

| **Sample** | **Haplotype**^a^ | **Haplogroup** |
| --- | --- | --- |
| NEA_001 | 126C-223T-278T-290T-310A-319A-362C | A2 |
| NEA_002 | 111T-223T-290T-319A-362C | A2 |
| NEA_003 | 111T-223T-290T-319A-362C | A2 |
| NEA_004 | 111T-223T-290T-319A-362C | A2 |
| NEA_005 | 126C-223T-278T-290T-319A-362C | A2 |
| NEA_006 | 126C-223T-278T-290T-319A-362C | A2 |
| NEA_007 | 111T-223T-290T-319A-362C-526A | A2 |
| NEA_008 | 111T-223T-290T-319A-362C | A2 |
| NEA_009 | 111T-223T-290T-319A-363C | A2 |
| NEA_010 | 111T-153A-223T-239A-290T-319A-362C-519C | A2 |
| NEA_011 | 104T-111T-223T-290T-319A-362C-519C | A2 |
| NEA_012 | 111T-223T-266T-290T-319A-362C | A2 |
| NEA_013 | 111T-223T-290T-319A-362C | A2 |
| NEA_014 | 126C-223T-278T-290T-319A-362C | A2 |
| NEA_015 | 111T-223T-290T-319A-362C | A2 |
| NEA_016 | 129A-223T-290T-319A-362C | A2 |
| NEA_017 | 111T-124C-220G-223T-290T-319A-362C | A2 |
| NEA_018 | 051G-111T-223T-267T-290T-293G-319A-362C | A2 |
| NEA_019 | 111T-223T-290T-319A-362C | A2 |
| NEA_020 | 111T-189C-223T-256T-290T-319A-362C | A2 |
| NEA_021 | 111T-126C-223T-258T-290T-319A-327T-362C | A2 |
| NEA_022 | 223T-290T-319A-362C-527C | A2 |
| NEA_023 | 092C-111T-223T-290T-319A-362C | A2 |
| NEA_024 | 111T-160T-223T-290T-319A-362C | A2 |
| NEA_025 | 126C-223T-278T-290T-319A-362C | A2 |
| NEA_026 | 111T-223T-290T-319A-362C | A2 |
| NEA_027 | 111T-223T-290T-319A-362C | A2 |
| NEA_028 | 051G-111T-223T-290T-319A-362C | A2 |
| NEA_029 | 084A-111T-215G-223T-290T-319A-362C | A2 |
| NEA_030 | 111T-189C | A2 |
| NEA_031 | 096T-111T-223T-290T-291T-319A-362C | A2 |
| NEA_032 | 111T-223T-266T-290T-319A-362C | A2 |
| NEA_033 | 111T-223T-290T-319A-362C-526A | A2 |
| NEA_034 | 111T-192T-223T-274A-319A-362C | A2 |
| NEA_035 | 096A-111T-290T-319A-362C | A2 |
| NEA_036 | 097C-098G-111T-223T-319A-320T-356C-362C | A2 |
| NEA_037 | 111T-290T-319A-362C | A2 |
| NEA_038 | 111T-223T-319A-362C | A2 |
| NEA_039 | 111T-129A-224C-290T-319A-362C | A2 |
| NEA_040 | 111T-129A-223T-224C-319A-362C | A2 |
| NEA_041 | 126C-223T-278T-290T-310A-319A-362C | A2 |
| NEA_042 | 104T-111T-185C-223T-290T-319A-362C | A2 |
| NEA_043 | 111T-189C-223T-290T-319A-362C | A2 |
| NEA_044 | 051G-111T-145A-189C | A2 |
| NEA_045 | 168T-182C-183C-189C-249C-312G-344T | B |
| NEA_046 | 092C-182C-183C-189C-217C-316G-359C | B |
| NEA_047 | 182C-183C-189C-217C-284G | B |
| NEA_048 | 092C-182C-183C-189C-217C-241G | B |
| NEA_049 | 168C-182C-183C-189C-217C-249C-312G | B |
| NEA_050 | 148T-182C-183C-188T-189C-217C-519C | B |
| NEA_051 | 096T-183C-188T-189C-217C-519C | B |
| NEA_052 | 172C-182C-183C-186T-189C-217C-519C | B |
| NEA_053 | 173T-182C-183C-189C | B |
| NEA_054 | 183C-189C | B |
| NEA_055 | 178C-183C-189C | B |
| NEA_056 | 092C-182C-183C-189C | B |
| NEA_057 | 153A-182C-183C-189C-217C-316G | B |
| NEA_058 | 16183C-16189C | B |
| NEA_059 | 169T-173T-182C-183C-189C-217C-357C | B |
| NEA_060 | 183C-189C-217C-316G | B |
| NEA_061 | 183C-189C-217C | B |
| NEA_062 | 183C-189C-217C-310A | B |
| NEA_063 | 182C-183C-189C-217C-311C | B |
| NEA_064 | 183C-189C-311C-217C | B |
| NEA_065 | 182C-183C-189C-217C-258C-362C-384A | B |
| NEA_066 | 223T-298C-325C-327T-362C | C1 |
| NEA_067 | 223T-298C-325C-327T-384A | C1 |
| NEA_068 | 223T-298C-325C-325T-526A | C1 |
| NEA_069 | 223T-298C-325C-327T | C1 |
| NEA_070 | 166G-193T-223T-249C-298C-325C-327T | C1 |
| NEA_071 | 154C-223T-298C-325C-327T-519C | C1 |
| NEA_072 | 126C-223T-270T-298C-325C-327T-438A | C1 |
| NEA_073 | 126C-223T-298C-325C-327T | C1 |
| NEA_074 | 051G-172C-223T-298C-325C-327T | C1 |
| NEA_075 | 223T-292T-298C-325C-327T-362C | C1 |
| NEA_076 | 051G-166G-223T-294T-298C-325C-327T-519C | C1 |
| NEA_077 | 051G-093C-223T-298C-325C-327T | C1 |
| NEA_078 | 173T-192T-223T-298C-325C-327T-346A-519C | C1 |
| NEA_079 | 223T-298C-325C-327T-519C | C1 |
| NEA_080 | 051G-172C-223T-298C-325C-327T | C1 |
| NEA_081 | 223T-298C-325C-327T-519C | C1 |
| NEA_082 | 051G-093C-223T-298C-325C-327T | C1 |
| NEA_083 | 223T-287T-298C-311C-325C-327T | C1 |
| NEA_084 | 092C-096A-223T-298C-325C-327T | C1 |
| NEA_085 | 223T-298C-325C-327T | C1 |
| NEA_086 | 223T-298C-325C-327T | C1 |
| NEA_087 | 223T-298C-319A-325C-327T | C1 |
| NEA_088 | 223T-298C-325C-327T-362C | C1 |
| NEA_089 | 154C-223T-298C-325C-327T | C1 |
| NEA_090 | 154C-223T-298C-325C-327T-519C | C1 |
| NEA_091 | 173C-192T-223T-298C-325C-327T-346A-519C | C1 |
| NEA_092 | 223T-295T-298C-319A-325C-327T | C1 |
| NEA_093 | 051G-172C-223T-298C-325C-327T | C1 |
| NEA_094 | 051G-172C-223T-295T-298C-325C-327T-335G | C1 |
| NEA_095 | 223T-298C-325C-327T | C1 |
| NEA_096 | 176T-223T-298C-311C-325C-327T | C1 |
| NEA_097 | 136C-223T-298C-325C-327T | C1 |
| NEA_098 | 051G-223T-266T-298C-C-327T | C1 |
| NEA_099 | 176T-223T-298C-311C-325C-327T | C1 |
| NEA_100 | 093C-192T-223T-298C-325C-327T | C1 |
| NEA_101 | 193T-223T-298C-325C-327T | C1 |
| NEA_102 | 223T-265C-298C-325C-327T-362C | C1 |
| NEA_103 | 223T-298C-325C-327T | C1 |
| NEA_104 | 126C-223T-298C-325C-327T | C1 |
| NEA_105 | 093C-223T-264T-287T-298C-311C-325C-327T | C1 |
| NEA_106 | 173T-192T-223T-298C-325C-327T-346A-519C | C1 |
| NEA_107 | 223T-270T-298C-325C-327T-456A | C1 |
| NEA_108 | 266G-298C-325C-327T-335G-362C | C1 |
| NEA_109 | 127G-223T-234T-279G-298C-310T-325C-327T | C1 |
| NEA_110 | 126C-172C-223T-270T-298C-325C-327T | C1 |
| NEA_111 | 051G-093C-223T-264T-287T-298C-311C-325C-327T | C1 |
| NEA_112 | 223T-298C-311C-325C-327T | C1 |
| NEA_113 | 176T-223T-298C-325C-327T | C1 |
| NEA_114 | 127G-223T-234T-298C-325C-327T | C1 |
| NEA_115 | 142T-179T-223T-295T-296T-325C-362C-497C-519C | D1 |
| NEA_116 | 223T-325C-362C | D1 |
| NEA_117 | 223T-325C-362C-519C | D1 |
| NEA_118 | 223T-325C-362C-519C | D1 |
| NEA_119 | 223T-163C-362C | D1 |
| NEA_120 | 142T-179T-223T-295T-325C-362C-497G-519C | D1 |
| NEA_121 | 142T-179T-223T-295T-325C-362C | D1 |
| NEA_122 | 223T-242T-16311C-325C-362C | D1 |
| NEA_123 | 223T-325C-362C | D1 |
| NEA_124 | 223T-325C-362C-519C | D1 |
| NEA_125 | 223T-325C-362C-519C | D1 |
| NEA_126 | 223T-325C-362C | D1 |
| NEA_127 | 187T-189C-209C-223T-325C-362C | D1 |
| NEA_128 | 142T-179T-295T-325C-362C-497G-519C | D1 |
| NEA_129 | 223T-362C-519C | D1 |
| NEA_130 | 172C-223T-239T-325C-362C | D1 |
| NEA_131 | 223T-224C-325C-362C-519C | D1 |
| NEA_132 | 142T-179T-223T-295T-325C-362C-497G-519C | D1 |
| NEA_133 | 114A-223T-241C-288C-301T-320T-362C | D1 |
| NEA_134 | 172C-223T-242T-311C-325C-362C | D1 |
| NEA_135 | 223T-242T-311C-325C-356C-362C | D1 |
| NEA_136 | 153A-298C | HV |
| NEA_137 | 259T-311C-519C | HV |
| NEA_138 | CRS | H |
| NEA_139 | 304C | H |
| NEA_140 | 519C | H |
| NEA_141 | 240G-309G | H |
| NEA_142 | 129A-519C | H |
| NEA_143 | 519C | H |
| NEA_144 | 124C-354T | H |
| NEA_145 | 293G | H |
| NEA_146 | CRS | H |
| NEA_147 | 259T-311C-519C | H |
| NEA_148 | 258T-311C | H |
| NEA_149 | 233G | H |
| NEA_150 | 519C | H |
| NEA_151 | 111C-209C-218T-519C | H |
| NEA_152 | 233G | H |
| NEA_153 | 233G | H |
| NEA_154 | 357C | H |
| NEA_155 | 311C-519C | H |
| NEA_156 | 114T | H |
| NEA_157 | 311C | H |
| NEA_158 | 162G-278T | H |
| NEA_159 | 093C-265G-519C | H |
| NEA_160 | 124C-354T-519C | H |
| NEA_161 | 124C-354T-519C | H |
| NEA_162 | 184T-519C | H |
| NEA_163 | 093C-162G-209C-320T-519C | H |
| NEA_164 | 093C-265G-519C | H |
| NEA_165 | 093C-145A | H |
| NEA_166 | 519C | H |
| NEA_167 | 093C-221T-311C-519C | H |
| NEA_168 | 069T-320T-519C | H |
| NEA_169 | 129A-223T -391A-519C | I |
| NEA_170 | 129A-172C-223T-311C-391A-519C | I |
| NEA_171 | 129A-172C-223T-311C-391A-519C | I |
| NEA_172 | 069T-092C-126C-261T-311C-519C | J |
| NEA_173 | 069T-126C | J |
| NEA_174 | 069t-126C | J |
| NEA_175 | 069T-126C-145A-172C-222T-260T-261T | J |
| NEA_176 | 069T-092C-126C-261T | J |
| NEA_177 | 063C-069T-126C | J |
| NEA_178 | 069T-126C-320T | J |
| NEA_179 | 223T-224C-234T-311C-519C | K |
| NEA_180 | 223T-225C-271T-311C | K |
| NEA_181 | 224C-311C-519C | K |
| NEA_182 | 148T-172C-187T-188G-189C-223T-230G-311C-320T-519C | L0 |
| NEA_183 | 126C-187C-189C-223T-264T-270T-278T-311C-519C | L1 |
| NEA_184 | 172C-187T-189C-223T-265C-278T-286G-294T-311C-360T-519C-527T | L1 |
| NEA_185 | 126C-187T-189C-223T-264T-270T-278T-293G-311C-400T-519C | L1 |
| NEA_186 | 223T-264T-278T-311C-390A | L2 |
| NEA_187 | 189C-192T-223T-278T-294T-309G-390A-519C | L2 |
| NEA_188 | 189C-192T-223T-278T-294A-309G-390A-519C | L2 |
| NEA_189 | 223T-320T-519C | L3 |
| NEA_190 | 172C-183C-189C-223T-311C-320T | L3 |
| NEA_191 | 124C-223T-256T-288C-368C | L3 |
| NEA_192 | 126C-235T-249T-293T-295T | T |
| NEA_193 | 126C-187C-294T-296T-519C | T |
| NEA_194 | 126C-163G-186T-189C-294T-519C | T |
| NEA_195 | 126C-294T-296T-304C-519C | T |
| NEA_196 | 096A-126C-294T-296T-304C-519C | T |
| NEA_197 | 051G-129C-183C-189C-356T-362C | U |
| NEA_198 | 096A-357C | U |
| NEA_199 | 265G-356C-362C-519C | U |
| NEA_200 | 051G-129C-183C-189C-362C | U |
| NEA_201 | 209C-342C | U |
| NEA_202 | 179T-320T-354T-356C-519C | U |
| NEA_203 | 192T-256T-270T-526A | U |
| NEA_204 | 256T-270T-399G | U |
| NEA_205 | 235T-298C | V |
| NEA_206 | 298C-519C | V |
| NEA_207 | 298C-519C | V |
| NEA_208 | 189C-197G-223T-278T-322G | X |
| NEA_209 | 126C-189A-223T-278T-519C | X |
| NEA_210 | 092C-111T-223T-290T-319A-362C-468C | A2 |
| NEA_211 | 092C-111T-223T-290T-319A-362C-468C | A2 |
| NEA_212 | 111T-223T-290T-319A-362C | A2 |
| NEA_213 | 111T-223T-290T-319A-362C | A2 |
| NEA_214 | 111T-223T-290T-319A-362C | A2 |
| NEA_215 | 111T-223T-290T-291T-319A-362C-399G | A2 |
| NEA_216 | 111T-223T-269G-290T-319A-362C | A2 |
| NEA_217 | 051G-111T-145A-223T-290T-293G-311C-319A-362C | A2 |
| NEA_218 | 111T-192T-223T-242T-290T-319A-362C-398A | A2 |
| NEA_219 | 178C-182C-189C-217C | B2 |
| NEA_220 | 092C-182C-183C-189C-217C-241C-304C | B2 |
| NEA_221 | 178C-183C-189C | B2 |
| NEA_222 | 183C-189C-217C-311C-319A | B2 |
| NEA_223 | 182C-183C-189C-217C-241C | B2 |
| NEA_224 | 183C-189C-217C | B2 |
| NEA_225 | 178C-183C-189C-217C | B2 |
| NEA_226 | 183C-189C-217C | B2 |
| NEA_227 | 183C-189C-217C | B2 |
| NEA_228 | 166G-178C-182C-183C-189C-217C-249C-312G-344T | B2 |
| NEA_229 | 183C-189C-217C | B2 |
| NEA_230 | 182C-189C | B2 |
| NEA_231 | 182C-189C-217C | B2 |
| NEA_232 | 183C-189C-217C-311C | B2 |
| NEA_233 | 168T-182C-183C-189C-217C-249C-312G | B2 |
| NEA_234 | 182C-183C-189C-217C-284G-316G-428A | B2 |
| NEA_235 | 183C-223T-248A-298C-325C-327T-360T | C1 |
| NEA_236 | 223T-298C-325C-327T | C1 |
| NEA_237 | 186C-223T-248A-298C-325C-327T-360T | C1 |
| NEA_238 | 126C-223T-270T-298C-325C-327T-438A | C1 |
| NEA_239 | 223T-298C-325C-327T | C1 |
| NEA_240 | 096A-136C-223T-298C-325C-327T | C1 |
| NEA_241 | 051G-172C-223T-295T-298C-325C-327T-335G | C1 |
| NEA_242 | 223T-248A-298C-325C-327T | C1 |
| NEA_243 | 051G-172C-223T-298C-325C-327T | C1 |
| NEA_244 | 223T-292T-298C-325C-327T-362C | C1 |
| NEA_245 | 223T-298C-325C-327T | C1 |
| NEA_246 | 189C-223T-325C-357C-362C | D1 |
| NEA_247 | 179T-223T-290T-319A-324C-362C | D1 |
| NEA_248 | 142T-179T-223T-295T-325C-362C | D1 |
| NEA_249 | 142T-179T-223T-295T-325C-362C | D1 |
| NEA_250 | 223T-239A-325C-362C | D1 |
| NEA_251 | 223T-311C-325C-362C-400T | D1 |
| NEA_252 | 223T-325C-362C | D1 |
| NEA_253 | 169insT-182C-183C-189C-192C-223T-249C-312G | H |
| NEA_254 | 079T-092C-224C-311C | K |
| NEA_255 | 129A-183C-189C-249C-311C | U |
| NEA_256 | 192T-256T-320T-322T-390A-399G | U |
| NEA_257 | 051G-075C-092C-129C-183C-189C-255A-362C | U |
| NEA_258 | 129A-148T-168T-172C-187C-188G-189C-223T-230G-311C-320T | L0 |
| NEA_259 | 129A-148T-168T-172C-187C-188G-189C-223T-230G-311C-320T | L0 |
| NEA_260 | 129A-148T-168T-172C-186T-188G-189C-223T-230G-311C-320T | L0 |
| NEA_261 | 223T-278T-294T-309G-390A | L2 |

^a^ SNPs positions are denoted using the CRS sequence as reference.
