# Supplementary material for: Mitochondrial DNA ancestry, HPV infection and the risk of cervical cancer in a multiethnic population of northeastern Argentina
Source: PLoS One. 2018 Jan 12;13(1):e0190966. doi: 10.1371/journal.pone.0190966 (PMC5766133; doi:10.1371/journal.pone.0190966)
Supplement: S2 Table — Legend: aO.R. adjusted by sample center location and nationality (Model II). Significant associations are in boldface. (DOCX) [file pone.0190966.s002.docx]

**S2 Table. Association analysis between mtDNA ancestry and cervical lesions.**

|  | O.R^a^ | CI 95% | *p* value |
| --- | --- | --- | --- |
| Ancestry |  |  |  |
| Amerindian | 1 | Ref |  |
| European | 1.4 | (0.8 – 2.7) | 0.240 |
| **African** | **3.8** | **(1.2 – 11.5)** | **0.018** |

Legend:  ^a^Ordered logistic regression for Pap cytology and mtDNA ancestry, adjusted by sample center location and nationality (Model II). Significant associations are in **boldface.**
