# Supplementary material for: Mitochondrial DNA ancestry, HPV infection and the risk of cervical cancer in a multiethnic population of northeastern Argentina
Source: PLoS One. 2018 Jan 12;13(1):e0190966. doi: 10.1371/journal.pone.0190966 (PMC5766133; doi:10.1371/journal.pone.0190966)
Supplement: S3 Table — Legend: aO.R. adjusted by sample center location and nationality (Model III). Significant associations are in boldface. (DOCX) [file pone.0190966.s003.docx]

**S3 Table. Association analysis between mtDNA haplogroups and cervical lesions.**

|  | O.R.^a^ | CI 95% | *p* value |
| --- | --- | --- | --- |
| Haplogroups^b^ |  |  |  |
| A | 1 | Ref |  |
| B | 1.6 | 0.6 – 4.2 | 0.349 |
| C | 0.7 | 0.3 – 1.6 | 0.378 |
| D | 0.8 | 0.3 – 2.3 | 0.668 |
| HV | 1.0 | 0.4 – 2.5 | 0.951 |
| JT | 2.5 | 0.7 – 8.5 | 0.149 |
| UK | 1.3 | 0.4 – 4.2 | 0.702 |
| **L** | **3.5** | **1.0 – 12.0** | **0.043** |

Legend: ^a^O.R. adjusted by sample center location and nationality (Model III). Significant associations are in **boldface.**
