# Supplementary material for: Mitochondrial DNA ancestry, HPV infection and the risk of cervical cancer in a multiethnic population of northeastern Argentina
Source: PLoS One. 2018 Jan 12;13(1):e0190966. doi: 10.1371/journal.pone.0190966 (PMC5766133; doi:10.1371/journal.pone.0190966)
Supplement: S4 Table — Legend: aO.R. adjusted by socio-demographic variables (Model IV). Significant associations are in boldface. bIncluding: HPV18, HPV59, HPV45 (specie A7). (DOCX) [file pone.0190966.s004.docx]

**S4 Table. Association analysis between Pap cytology, mtDNA ancestry and HPV infection.**

|  | O.R.^a^ | CI 95% | *p* value |
| --- | --- | --- | --- |
| Ancestry |  |  |  |
| Amerindian | 1 | Ref |  |
| European | 1.7 | 0.9 – 3.3 | 0.122 |
| African | 3.2 | 0.8 – 12.2 | 0.085 |
|  |  |  |  |
| HPV types |  |  |  |
| **16** | **24.2** | **9.3 – 62.7** | **<0.001** |
| **33** | **5.5** | **1.2 – 24.5** | **0.026** |
| 52 | 1.6 | 0.2 – 16.3 | 0.671 |
| 56 | 1.9 | 0.4 – 8.3 | 0.412 |
| **58** | **19.0** | **2.4 – 147.7** | **0.005** |
| **6/11** | **6.6** | **1.9 – 22.9** | **0.003** |
| **Other HPV-HR^b^** | **6.3** | 1.2 – 24.5 | **0.011** |
| **Multiple-Infections** | **4.2** | **1.8 – 9.8** | **0.001** |
| HPV-Undetermined | 1.6 | 0.5 – 5.2 | 0.391 |

Legend: ^a^O.R. adjusted by socio-demographic variables (Model IV). Significant associations are in **bold.** ^b^Including: HPV18, HPV59, HPV45 (specie *A7*)
