# Supplementary material for: Mitochondrial DNA ancestry, HPV infection and the risk of cervical cancer in a multiethnic population of northeastern Argentina
Source: PLoS One. 2018 Jan 12;13(1):e0190966. doi: 10.1371/journal.pone.0190966 (PMC5766133; doi:10.1371/journal.pone.0190966)
Supplement: S5 Table — Legend: O.R. adjusted by socio-demographic variables (Model V). Significant associations are shown in boldface. (DOCX) [file pone.0190966.s005.docx]

**S5 Table. Association analysis between Pap cytology, mtDNA haplogroups and HPV infection.**

|  | O.R.^a^ | CI 95% | *p* value |
| --- | --- | --- | --- |
| Haplogroups |  |  |  |
| A | 1 | Ref | - |
| B | 1.7 | 0.6 – 5.1 | 0.345 |
| C | 0.5 | 0.2 – 1.2 | 0.130 |
| D | 0.7 | 0.2 – 2.3 | 0.583 |
| HV | 1.0 | 0.4 – 2.8 | 0.924 |
| JT | 2.2 | 0.6 – 8.2 | 0.252 |
| UK | 1.5 | 0.4 – 5.0 | 0.527 |
| L | 2.5 | 0.6 – 10.7 | 0.229 |
|  |  |  |  |
| HPV types |  |  |  |
| **16** | **25.5** | **9.6 – 68.1** | **< 0.001** |
| **33** | **4.9** | **1.1 – 22.3** | **0.041** |
| 52 | 1.0 | 0.1 – 10.9 | 0.982 |
| 56 | 1.6 | 0.4 – 7.3 | 0.506 |
| **58** | **11.8** | **1.4 – 100.9** | **0.024** |
| **6/11** | **7.8** | **2.0 – 30.7** | **0.003** |
| **Other HPV-HR^b^** | **7.4** | **1.5 – 35.3** | **0.012** |
| **Multiple-Infections** | **3.9** | **1.6 – 9.2** | **0.002** |
| HPV-Undetermined | 1.7 | 0.5 – 5.3 | 0.388 |

Legend: ^a^O.R. adjusted by socio-demographic variables (Model V). Significant associations are shown in boldface.
